# Diacylglycerol Kinase Zeta Positively Controls the Development of *i*NKT-17 Cells

**DOI:** 10.1371/journal.pone.0075202

**Published:** 2013-09-20

**Authors:** Jinhong Wu, Shudan Shen, Jialong Yang, Zhenwei Xia, Xiao-Ping Zhong

**Affiliations:** 1 Department of Pediatrics, Division of Allergy and Immunology, Duke University Medical Center, Durham, North Carolina, United States of America; 2 Department of Pediatrics, Ruijin Hospital, Shanghai Jiao Tong University School of Medicine, Shanghai, China; 3 Department of Immunology, Duke University Medical Center, Durham, North Carolina, United States of America; Université Libre de Bruxelles, Belgium

## Abstract

Invariant natural killer T (iNKT) cells play important roles in bridging innate and adaptive immunity via rapidly producing a variety of cytokines. A small subset of *i*NKT cells produces IL-17 and is generated in the thymus during *i*NKT-cell ontogeny. The mechanisms that control the development of these IL-17-producing *i*NKT-17 cells (*i*NKT-17) are still not well defined. Diacylglycerol kinase ζ (DGKζ) belongs to a family of enzymes that catalyze the phosphorylation and conversion of diacylglycerol to phosphatidic acid, two important second messengers involved in signaling from numerous receptors. We report here that DGKζ plays an important role in *i*NKT-17 development. A deficiency of DGKζ in mice causes a significant reduction of *i*NKT-17 cells, which is correlated with decreased RORγt and IL-23 receptor expression. Interestingly, *i*NKT-17 defects caused by DGKζ deficiency can be corrected in chimeric mice reconstituted with mixed wild-type and DGKζ-deficient bone marrow cells. Taken together, our data identify DGKζ as an important regulator of *i*NKT-17 development through *i*NKT-cell extrinsic mechanisms.

## Introduction

Invariant natural killer T (iNKT) cells represent a unique T-cell lineage with the ability to bridge innate and adaptive immune responses [1-4]. *i*NKT cells express the invariant Vα14-Jα18 TCR (*i*Vα14TCR) in mice and the Vα24-Jα18 TCR in humans, with limited TCRVβ usages. *i*NKT cells are positively selected in the thymus after the engagement of the *i*Vα14TCR with glycolipids presented by CD1d expressed on CD4^+^CD8^+^ double-positive (DP) thymocytes [5-8]. Post-selected *i*NKT cells undergo defined developmental stages, including stage 1 (CD44^-^NK1.1^-^), stage 2 (CD44^+^NK1.1^-^), and terminally differentiated stage 3 (CD44^+^NK1.1^+^) [5,6,9,10]. Different from conventional αβ T cells, *i*NKT cells rapidly produce copious amounts of cytokines such as IL-4, IFNγ, and TNFα following stimulation of the *i*Vα14TCR with agonist ligands, such as α-galactosylceramide (α-GalCer) and endogenous and microbial ligands [11-14].

Recently, *i*NKT cells capable of producing the IL-17 family of cytokines (*i*NKT-17), such as IL-17A, IL-17F, and IL-22, have been identified [15-18]. *i*NKT cell-derived IL-17-family cytokines are implicated in both inflammatory responses such as airway inflammation via recruiting neutrophils and protective roles such as suppression of liver inflammation [19,20]. *i*NKT-17 cells are generated in the thymus and are considered to be developmentally programmed [17,21]. *i*NKT-17 cells are mainly restricted to the NK1.1^-^ CD4^-^ population [15] and express the marker for recent thymic emigrant and nature-regulatory T cells neuropilin-1 [16]. Additionally, *i*NKT-17 cells express molecules that are usually characteristic of Th17 cells such as the orphan nuclear receptor RORγt, the IL-23 receptor (IL-23R), and the chemokine receptor CCR6 [17,22,23]. Although it has become clear that *i*NKT-17 represents a unique *i*NKT sublineage with important functions in the pathogenesis of diseases, the signal control for the generation/maintenance of this sublineage of *i*NKT cells is not well understood.

Diacyglcerol kinase ζ (DGKζ) belongs to a family of 10 enzymes that phosphorylate diacylglycerol (DAG) to produce phosphatidic acid (PA), two important second messengers involved in signaling from numerous receptors [24-26]. DGKζ is expressed in many cell lineages in the immune system, such as T cells, macrophages, dendritic cells, and mast cells [27-30]. Recent studies have demonstrated that DGK activity plays important regulatory roles in these immune-cell lineages via terminating DAG and simultaneously generating PA [28,29,31]. In T cells, DGKζ negatively controls TCR-induced activation of the RasGRP1-Ras-Erk1/2 pathway, the PKCθ-NFκB pathway, and mTOR signaling [27,30,32,33], inhibits T cell activation *in vitro* and *in vivo* [27,30], inhibits primary anti-viral immune responses but promotes memory CD8 T-cell-mediated anti-viral immune responses [34], contributes to T-cell anergy and tumor evasion [31], and, together with DGKα, promotes the positive selection of conventional αβ T (cαβT) cells [35]. DGKζ has also been demonstrated to regulate TLR signaling and the production of proinflammatory cytokines such as IL-12p40 and TNFα to control innate and adaptive immune responses to parasite infection [26] and to modulate mast-cell survival and activation [29]. Recently, we have demonstrated that deficiency of both DGKζ and α, another isoform expressed in T cells, causes severe decreases of *i*NKT cells in mice [33]. However, deficiency of either DGKα or DGKζ alone does not result in a noticeable abnormality of *i*NKT-cell numbers in mice. In this report, we demonstrate that germline deficiency of DGKζ leads to decreases of IL-17 producing *i*NKT cells without an obvious effect on IL-4- and IFNγ-producing *i*NKT (*i*NKT-4 and -1) cells. The decrease of *i*NKT-17 cells caused by DGKζ deficiency is correlated with a reduced expression of RORγt and IL-23R. Interestingly, in chimeric mice reconstituted with mixed WT and DGKζ bone marrow (BM) cells, an *i*NKT-17 defect caused by DGKζ deficiency can be corrected, suggesting that DGKζ controls *i*NKT-17 development via *i*NKT extrinsic mechanisms.

## Methods

### Mice and cells

DGKζ-deficient (DGKζKO) mice backcrossed to C57BL/6J background for at least nine generations were previously reported [27,31]. C57BL/6J and CD45.1^+^ congenic mice were generated by in-house breeding. TCRαKO mice were purchased from the Jackson Laboratory. All mice were housed in a pathogen-free facility. This study was carried out in strict accordance with the recommendations in the *Guide for the Care and Use of Laboratory Animals of the National Institutes of Health*. All mice were used according to protocols approved by the Institutional Animal Care and Use Committee of Duke University (Protocol Number: A132-10-5). Splenocytes, thymocytes, and liver MNCs were made according to previously published protocols [33,36].

### Antibodies and flow cytometry

Iscove’s Modified Dulbecco’s Medium (IMDM) was supplemented with 10% (vol/vol) FBS, penicillin/streptomycin, and 50 µM 2-mercaptoethanol (IMDM-10). PE- or APC-conjugated mouse CD1d tetramers loaded with PBS-57 were provided by the NIH Tetramer Facility. The Live/Dead^®^ Fixable Violet Dead Cell Stain Kit was purchased from Invitrogen. Fluorescence-conjugated anti-mouse TCRβ (H57-597), CD45.1 (A20), Thy1.2 (30-H12), IFNγ (XMG1.2), IL-17A (TC11-18 H10.1), IL-4 (11B11), and RORγt (ATKJS-9) antibodies were purchased from BioLegend.

Cell-surface staining was performed with 2% FBS-PBS. Intracellular staining for IFNγ, IL-17A, and IL-4 was performed using BD Biosciences Cytofix/Cytoperm^™^ and perm/wash solutions following the manufacturer’s protocol. All flow cytometry data were collected using FACS Canto-II (BD Biosciences) and analyzed with the FlowJo software. A solution of 0.5% Tween-20-PBS was used to dissolve α-GalCer (Enzo life science).

### Purification of *i*NKT cells and real-time quantitative PCR

*i*NKT cells were enriched with PE-CD1dTet and anti-PE-MACS-beads according to a previously published protocol [33,36]. Enriched *i*NKT cells were stained with anti-TCRβ and 7-AAD and sorted for live CD1dTet ^+^ TCRβ^+^ *i*NKT cells with greater than 98% purity using MoFlo. Sorted *i*NKT cells were immediately lysed in Trizol for RNA preparation. cDNA was made using the iScript Select cDNA Synthesis Kit (Biorad). Real-time quantitative PCR was conducted and analyzed as previously described [33,36]. Expressed levels of target mRNAs were normalized with β-actin and calculated using the 2^–ΔΔCT^ method. Primers were as follows: IL-23R, Forward: 5’-AGCAAAATCATCCCACGAAC-3’, Reverse: 5’- GAAGACCATTCCCGACAAAA-3’; RORc, Forward: 5’-CGACTGGAGGACCTTCTACG-3’, Reverse: 5’-TTGGCAAACTCCACCACATA-3’; IFN-γ, Forward: 5’-GCGTCATTGAATCACACCTG-3’, Reverse: 5’-TGAGCTCATTGAATGCTTGG-3’; IL-4, Forward: 5’-ACAGGAGAAGGGACGCCAT-3’, Reverse: 5’-GAAGCCCTACAGACGAGCTCA-3’; IL-17A, Forward: 5’-GCTCCACAAGGCCCTCAGA-3’, Reverse: 5’-CTTTCCCTCCGCATTGACA-3’; DGK-α, Forward: 5’-GATGCAGGCACCCTGTACAAT-3’, Reverse: 5’-GGACCCATAAGCATAGGCATCT-3’; DGK-ζ, Forward: 5’-CTGAGGAGCAGATCCAGA GC-3’; DGK-δ, Forward: 5’-GATCCTCGAGCCTCTGCGTTCTCTGC-3’, Reverse: 5’-GATCGCGGCCGCGGCCAGAACACAT-3’.

### In vitro stimulation of *i*NKT cells

For α-GalCer stimulation, 1 x 10^7^ thymocytes, 5 x 10^6^ splenocytes, or 5 x 10^6^ lymph node (LN) cells were seeded in a 48-well plate in 1 ml IMDM-10 or 5 x 10^5^ liver MNCs were seeded in a 96-well plate in 200 µl IMDM-10. Cells were left unstimulated or stimulated with α-GalCer (125 ng/ml) for 72 hours with the addition of PMA (50 ng/ml) and ionomycin (500 ng/ml) and GolgiPlug™ (1ng/ml) in the last 5 hours. For short-term PMA plus ionomycin stimulation, 0.5-1 x 10^6^ enriched *i*NKT cells from thymocytes and splenocytes or density-enriched liver MNCs were seeded in a 96-well V-bottom plate in 200 µl IMDM-10. Cells were stimulated with PMA plus ionomycin for 5 hours in the presence of GolgiPlug™. After stimulation, cells were first stained with CD1dTet, anti-TCRβ, anti-Lin (B220, Gr1, CD1b, CD11c, and CD8), and Live/Dead followed by intracellular staining for IFNγ, IL-17A, IL-4, and RORγt. *i*NKT cells were gated on live B220^-^, Gr1^-^, CD11b^-^, CD11c^-^, and CD8^-^ cells.

### In vivo stimulation of *i*NKT cells

Mice were intraperitoneally injected with 150 µg Brefeldin A in 100 µl. Ninety minutes later, mice were intraperitoneally injected with 2 µg α-GalCer diluted in 200 µl PBS. Two hours after the α-GalCer injection, splenocytes and liver MNCs were intracellularly stained for IFNγ, IL-4, and IL-17A. Total RNA from splenocytes was also isolated from mice injected with α-GalCer without a Brefeldin A pretreatment.

### Bone marrow chimeric mice

*TCRα* ^*-/-*^ mice were sublethally irradiated (600 rad) and intravenously injected with a mixture of WT (CD45.1^+^) and DGKζKO (CD45.2^+^) BM cells at a 1:2 ratio. Thymocytes and splenocytes from the recipient mice were harvested 8 weeks later.

### Statistical analysis

Data are presented as mean ± SEM and statistical significance were determined by a Student’s *t*-test.

## Results

### *DGKζ* deficiency does not affect *i*NKT cell proliferation *in vitro*

**Figure 1:**
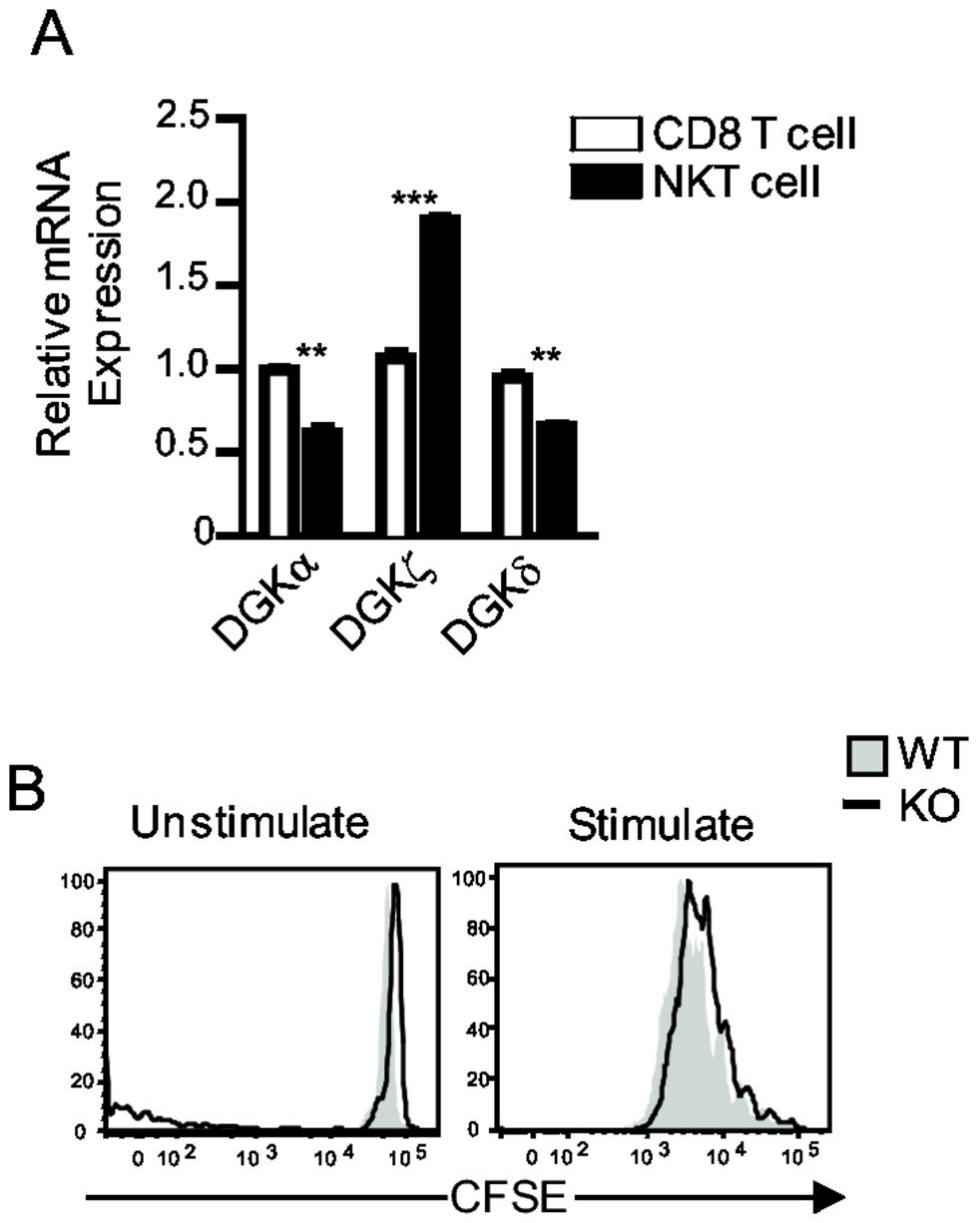
DGKζ is dispensable for *i*NKT cell activation in vitro. (**A**) Quantitative real-time PCR analysis of DGK-α, ζ and δ mRNA in sorted CD8 cαβT cells and *i*NKT cells from wild-type mice. Data are representative of three independent experiments. **, *P* < 0.01, and ***, *P* < 0.001 determined unpaired two-tail Student *t*-test. (**B**) *i*NKT cell proliferation assessed by CFSE-dilution assay. CFSE-labeled WT and DGKζKO thymoyctes were left unstimulated or stimuated with α-Galcer in vitro for 72 hours. Overlaid histograms show CFSE intensity in live gated CD1dTet ^+^ TCRβ^+^ *i*NKT cells. Data shown represent three experiments.

DGKα, ζ, and δ are the dominant isoforms that expressed in T cells [25,31]. We compared the expression of these isoforms between cαβT cells and *i*NKT cells. As shown in Figure 1A, both DGKα and δ were expressed at reduced levels in *i*NKT cells compared with CD8^+^ cαβT cells. However, DGKζ was expressed at a higher level in *i*NKT cells than in CD8^+^ T cells. The reason for the differential expression of DGK isoforms between cαβT and *i*NKT cells remains to be defined.

Previously, studies have demonstrated that a deficiency of DGKζ does not affect *i*NKT-cell development. The total numbers and developmental stages of *i*NKT cells in DGKζKO mice are not obviously different from WT control mice [33]. To examine whether DGKζ regulates *i*NKT-cell activation *in vitro*, we labeled WT and DGKζ deficient thymocytes with CFSE and then stimulated the cells with α-GalCer in vitro for 72 hours. As shown in Figure 1B, DGKζKO and WT *i*NKT cells expanded and proliferated similarly, suggesting that DGKζ deficiency did not affect TCR-induced *i*NKT- cell proliferative response *in vitro*. It has been demonstrated that DGKζKO cαβT cells are hyperproliferative in response to TCR stimulation [27]. Thus, DGKζ differentially controls cαβT and *i*NKT-cell proliferation *in vitro*.

### Decreased IL-17 but not IFNγ or IL-4 production by *DGKζ* deficient *i*NKT cells following in vitro stimulation of the *i*Vα14TCR

**Figure 2:**
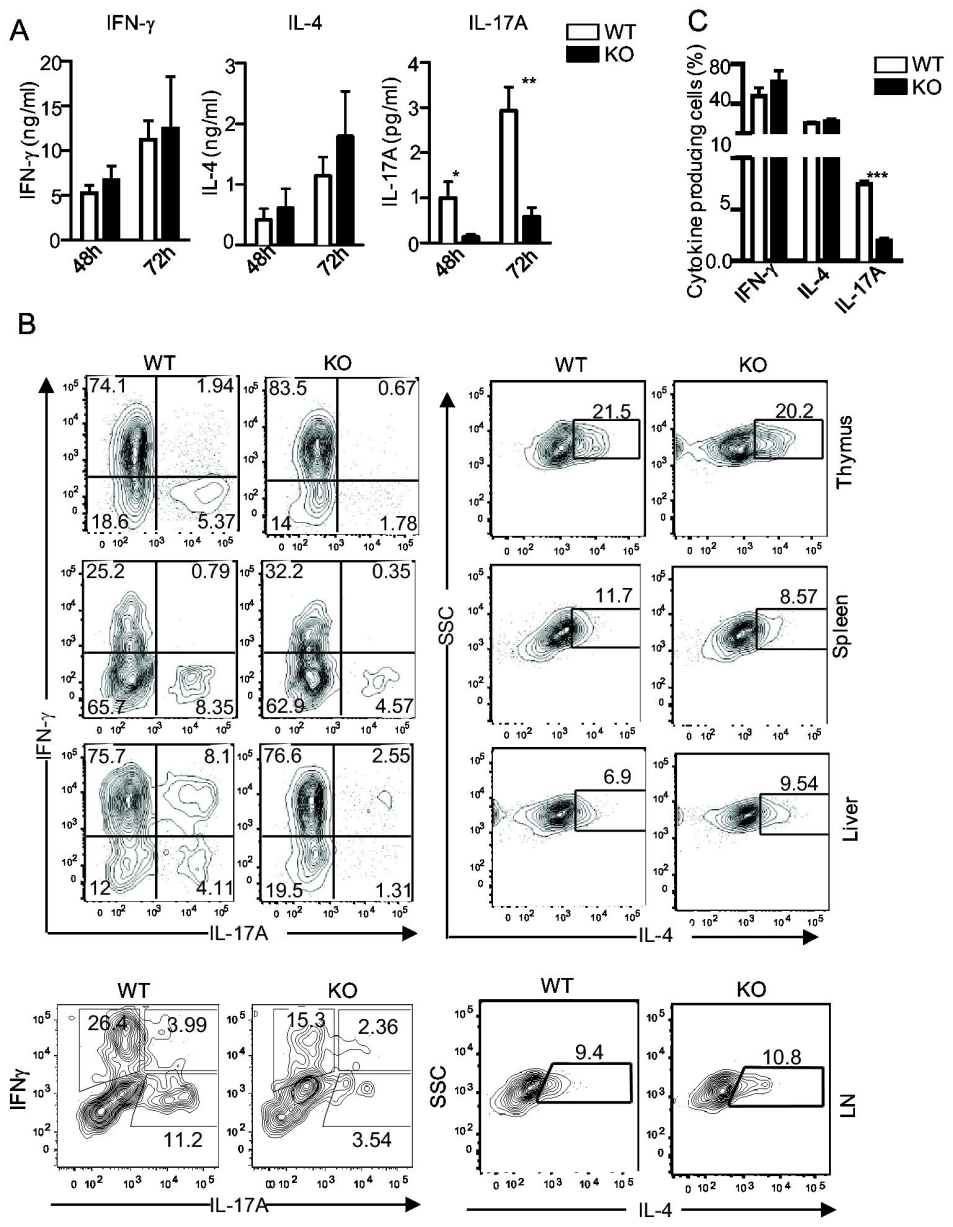
Decreased IL-17A production by DGKζ deficient *i*NKT cells following α-GalCer stimulation in vitro. Thymocytes, splenocytes, and liver mononuclear cells (MNC) from WT, *DGKζ* ^*-/-*^ mice were stimulated with α-GalCer for 72 hours *in* *vitro*. (**A**) IL-4, IFNγ, and IL-17A levels in culture supernatants of thymocytes measured by ELISA. (**B**) Intracellular staining of IL-4, IFNγ, and IL-17A of thymic, splenic, liver, and lymph node (LN) *i*NKT cells stimulated with α-GalCer for 72 hours with PMA and ionomycin in the presence of GolgiPlug during the last 5 hours of culture. (**C**) Percentages of thymic *i*NKT cells producing the indicated cytokines. Bar graphs are mean ± SEM calculated from three experiments.

*i*NKT cells produce multiple cytokines to regulate immune responses. To determine whether DGKζ regulates cytokine production by *i*NKT cells during *in vitro* activation, we stimulated WT and DGKζKO thymocytes with α-GalCer for 48 and 72 hours; IFNγ, IL-4, and IL-17 levels in culture supernatants were measured by ELISA. No obvious differences of IFNγ and IL-4 levels were observed between WT and DGKζKO *i*NKT cells. In contrast, IL-17A levels were considerably decreased in DGKζ *i*NKT cells (Figure 2A). Consistent with these ELISA data, intracellular staining of these cytokines in *i*NKT cells also showed decreased IL-17A but similar IFNγ- and IL-4-producing *i*NKT cells following α-GalCer stimulation (Figure 2B and 2C). Taken together, these data indicate that DGKζ plays an important role for IL-17 production by *i*NKT cells *in vitro*.

### Impaired *i*NKT-17 development in the absence of DGKζ

**Figure 3:**
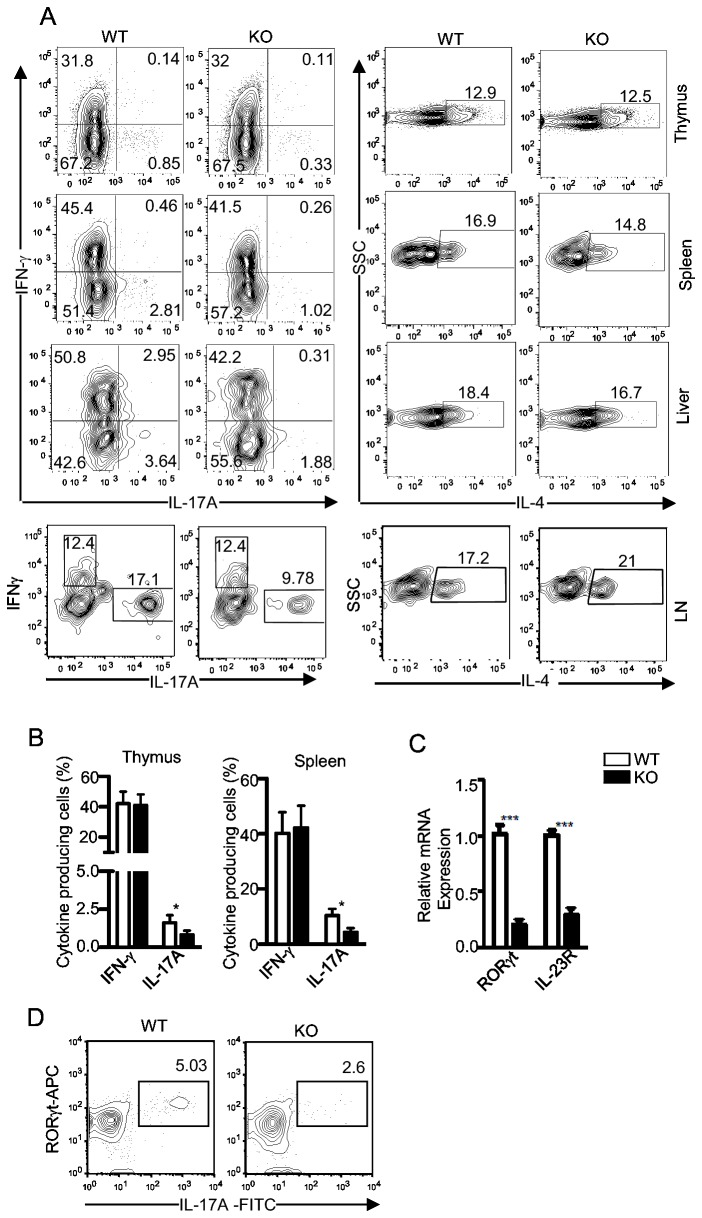
*i*NKT-17 developmental defect in the absence of DGKζ. (**A**,**B**) *i*NKT-cells enriched from WT and DGKζKO thymocytes, splenocytes, liver mononuclear cells, and LN cells were stimulated with PMA and ionomycin for 5 hours in the presence of Golgi-Plug. Contour plots show intracellular staining of indicated cytokines in gated CD1dTet ^+^ TCRβ ^+^ Lin^-^(Gr1^-^B220^-^CD8^-^CD11c^-^CD11b^-^) *i*NKT cells (A). Bar graph (B) represents mean ± SEM of percentages of indicated cytokines in gated *i*NKT-cells (n=3). (**C**) Decreased RORγt and IL23R expression in DGKζKO *i*NKT cells. RORγt and IL23R mRNA levels in sorted thymi *i*NKT cells from WT and DGKζKO mice were measured by quantitative real-time PCR. *, *P*<0.05; ***, *P* < 0.001 (*t*-test). (**D**) Co-intracellular staining of IL-17A and RORγt in thymic WT and DGKζKO *i*NKT cells following PMA + ionomycin stimulation for 5 hours. Data shown are representative or calculated from three experiments.

The impaired production of IL-17A by *i*NKT cells following α-GalCer stimulation can be caused by a developmental defect or impaired expansion of *i*NKT-17 cells. To determine whether DGKζ deficiency causes a developmental defect in generating *i*NKT-17 cells, we enriched *i*NKT cells from WT and DGKζKO thymocytes and stimulated enriched *i*NKT cells with PMA plus ionomycin *in vitro* for 5 hours in the presence of GolgiPlug. Intracellular staining of cytokines showed decreased IL-17A positive cells within DGKζKO *i*NKT cells than in WT controls (Figure 3A and 3B). In contrast, the percentages of IFNγ- and IL-4-producing cells were similar in WT and DGKζKO *i*NKT cells. RORγt and IL-23R signaling is critical for the *i*NKT-17 differentiation [17,22]. We sorted *i*NKT cells from WT and DGK-ζKO thymocytes and measured RORγt and IL-23R mRNA levels by quantitative real-time PCR. Consistent with the *i*NKT-17 developmental defect, IL-23R and RORγt mRNA levels were obviously decreased in DGKζKO *i*NKT cells compared with WT *i*NKT cells (Figure 3C). Consistent with these observations, DGKζKO thymic *i*NKT cells contained much less IL-17A ^+^ RORγ^+^ double positive cells than WT controls (Figure 3D). Together, these results suggest that DGKζ at least promotes *i*NKT-17 differentiation during development.

### Impaired *in vivo* IL-17 induction in DGKζ deficiency mice following α-GalCer treatment

**Figure 4:**
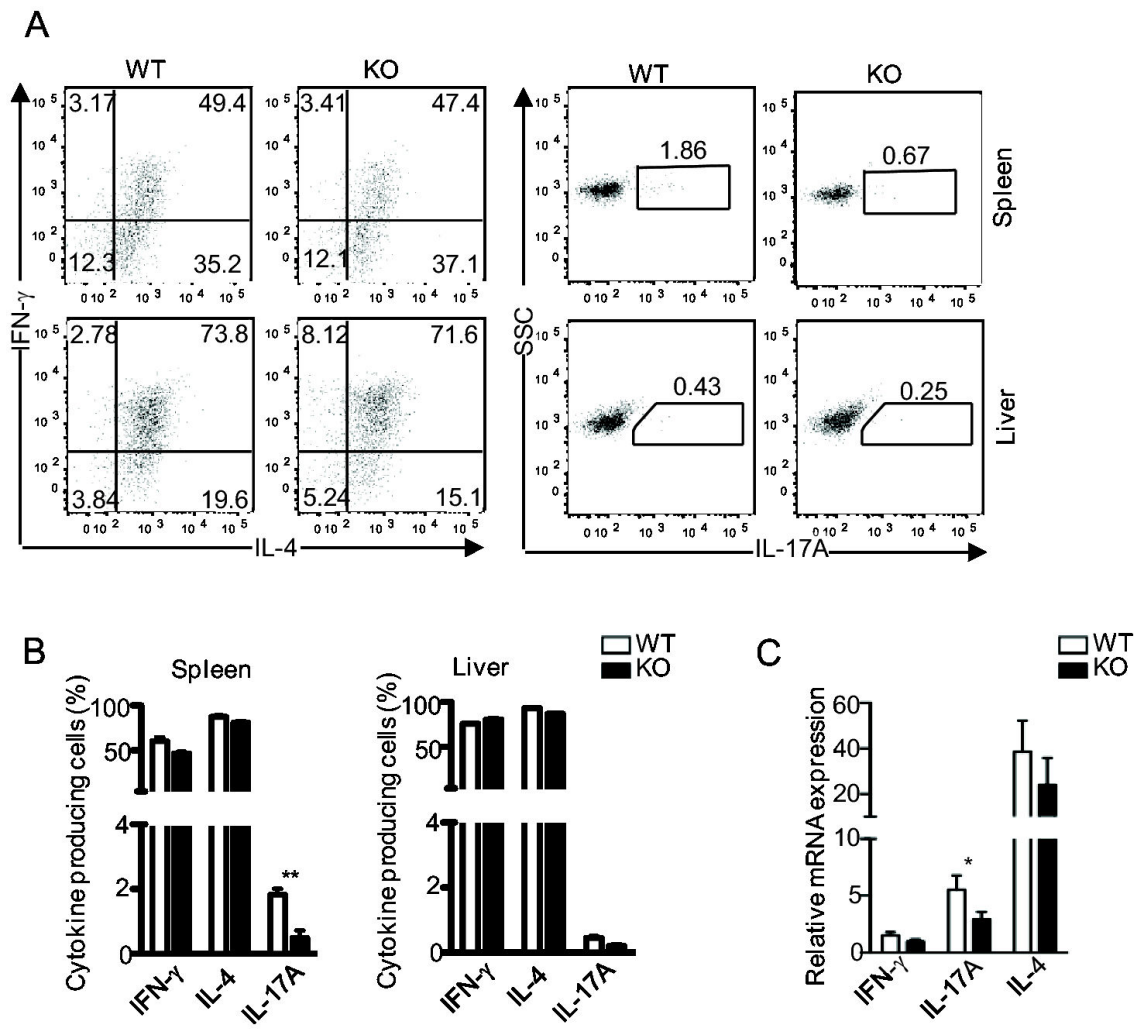
Decreased IL-17A expression following *i*NKT cell activation *in vivo*. WT and DGKζKO mice were intraperitoneally injected with 150µg brefeldin A. Ninety minutes later, mice were intraperitoneally injected with 2 µg α-GalCer diluted in 200 µl PBS. Two hours after α-GalCer injection, IFN-γ, IL-4, and IL-17A positive *i*NKT cells in the spleen and liver were determined by flow-cytometry. (**A**) Representative dot plots show intracellularly stained cytokines in gated *i*NKT cells expression. (**B**) Mean ± SEM presentation of *i*NKT cells expressing the indicated cytokines. (**C**) Decreased IL-17A mRNA in DGKζKO spleen 4 hours after α-GalCer injection. Data shown represent two experiments.

The data shown above reveal the important role of DGKζ of IL-17 production *in vitro*. We further examined how DGKζ deficiency may affect *i*NKT-cell cytokine production *in vivo*. As shown in Figure 4A and 4B, intracellular staining showed that the percentages of IL-4 or IFNγ positive *i*NKT cells were similar between WT and DGKζKO mice 2 hours after the α-GalCer injection. However, the percentage of IL-17-producing *i*NKT cells was obviously lower in DGKζKO mice than in WT mice. Moreover, the IL-17A mRNA level, although not IL-4 or IFNγ mRNA levels, was decreased in the DGKζKO spleen after the α-GalCer injection (Figure 4C). Together, these observations suggest that DGKζ is important for optimal IL-17 expression in *i*NKT cells *in vivo*.

### Promotion of *i*NKT-17 differentiation by DGKζ is not *i*NKT cell intrinsic

**Figure 5:**
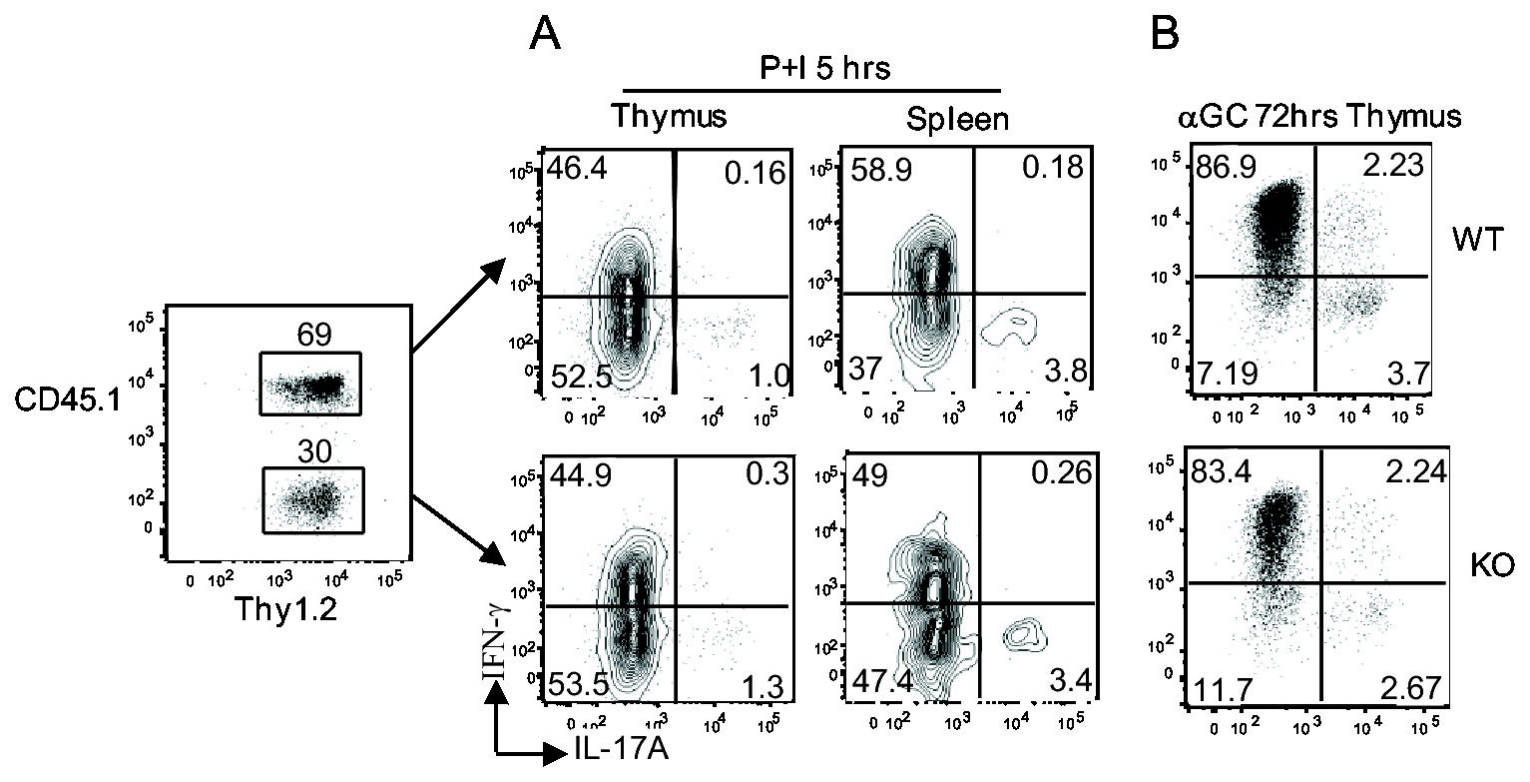
*i*NKT-17 developmental defect in DGKζ deficiency mice is due to cell extrinsic mechanism. Sublethally irradiated *TCRα* ^*-/-*^ mice were i.v. injected with WT (CD45.1) and DGKζ KO (CD45.2) BM cells at a 1:2 ratio. (**A**) Enriched *i*NKT-cells from thymocytes or splenocytes from chimeric mice stimulated with PMA and Ionomycin for 5 hours in the presence of a GolgiPlug. Intracellular IL-17 and IFNγ staining in WT and DGKζ KO *i*NKT-cells were gated in *i*NKT-cells. (**B**) Ten million WT and DGK-ζ KO thymocytes stimulated with α-GalCer for 72 hours. Intracellular IL-17 and IFNγ staining in WT and DGKζ KO *i*NKT-cells were gated in *i*NKT-cells. Data shown are representative of three chimeras from two independent experiments.

Because DGKζ was deficient in all cell lineages in DGKζKO mice, the aforementioned *i*NKT-17 defect in these mice could be caused by extrinsic or intrinsic mechanisms. To distinguish these possibilities, we generated mixed-bone-marrow chimeric mice by co-injecting CD45.1^+^ WT and CD45.2^+^ DGKζKO BM cells at a 1:2 ratio into sublethally irradiated *TCRα* ^*-/-*^ mice. Eight weeks after reconstitution, *i*NKT cells from thymocytes or splenocytes of the chimeric mice were enriched and stimulated with PMA plus ionomycin for 5 hours or stimulated with α-GalCer for 72 hours to induce IL-17 and IFNγ production. As shown in Figure 5, similar percentages of DGKζKO and WT *i*NKT cells produced IL-17A, suggesting that the impairment of *i*NKT-17 differentiation caused by DGKζ deficiency likely resulted from mechanisms extrinsic to *i*NKT cells.

## Discussion

In this report, we demonstrated that DGKζ plays a selective role in promoting *i*NKT-17 development. We have shown that a deficiency of DGKζ resulted in impaired *i*NKT-17 correlated with decreased expression of RORγt and IL-23R. In contrast, IFNγ-producing *i*NKT-1 or IL-4-producing *i*NKT-4 cell development seemed not to be affected by DGKζ activity.

At least three DGK isoforms, α, δ, and ζ, are expressed in *i*NKT cells. While sharing common structural features such as the kinase domain and the cysteine-rich C1 domains, they also contain distinct structural domains/motifs and belong to different subtypes of the DGK family [37]. We have demonstrated that DGKα and ζ function synergistically to promote *i*NKT-cell development/homeostasis and cαβ T cell maturation [33,35]. Additionally, deficiency of either DGKα or ζ results in enhanced activation of cαβT-cell activation reflected by hyper-proliferation and elevated cytokine production [27,31]. However, DGKζ deficiency does not obviously impact *i*NKT cell activation. DGKζ-deficient *i*NKT cells proliferate and secrete IFNγ and IL-4 similarly to WT *i*NKT cells following TCR engagement. Thus, *i*NKT cells and cαβT cells display a differential requirement of DGKζ for modulating their activation. At present, we cannot rule out that DGKα or δ may function redundantly with DGKζ in the control of *i*NKT cell activation. The virtual absence of *i*NKT cells in DGKα and ζ double-deficient mice prevents us from addressing this issue. Further generation and analysis of mice with conditional ablation of multiple DGK isoforms in mature *i*NKT cells should provide a solid conclusion regarding the role of DGK activity in *i*NKT cell activation.

Our data indicate that DGKζ promotes *i*NKT-17 differentiation via *i*NKT-extrinsic mechanisms. Important questions remain to be addressed about which cell lineage DGKζ controls *i*NKT-17 differentiation and how DGKζ exerts such functions in this cell lineage. *i*NKT-17 development is intrinsically dependent on RORγt but is negatively controlled by Th-POK, a transcript factor critical for CD4 lineage development [17,21,38,39]. Extracellular factors such as IL-23 and IL-1 are indispensable for *i*NKT-17 differentiation [22,40]. Interestingly, we have found that DGKζ is important for IL-12p40 expression in macrophages and dendritic cells [28]. A decrease of expression of IL-12p40, a subunit for both IL-12 and IL-23, could potentially lead to impaired *i*NKT-17 differentiation. Additionally, DGK activity inhibits mTOR activation in T cells [32]. mTOR activity can negatively control IL-12p40 transcription in dendritic cells and macrophages [41-44]. Thus, it is possible that a potential elevation of mTOR activity in dendritic cells may cause down-regulation of IL-23 expression by dendritic cells, leading to impaired *i*NKT-17 differentiation. Future studies using DGKζ conditional knockout mice should help to identify the lineage in which, and the mechanisms by which, DGKζ functions to promote *i*NKT-17 differentiation.
